# Practical Solutions to Address COVID-19-Related Mental and Physical Health Challenges Among Low-Income Older Adults

**DOI:** 10.3389/fpubh.2021.674847

**Published:** 2021-07-12

**Authors:** Caroline D. Bergeron, Ali Boolani, Erica C. Jansen, Matthew Lee Smith

**Affiliations:** ^1^Public Health Agency of Canada, Division of Aging, Seniors and Dementia, Ottawa, ON, Canada; ^2^Center for Population Health and Aging, Texas A&M University, College Station, TX, United States; ^3^Department of Physical Therapy, Clarkson University, Potsdam, NY, United States; ^4^Department of Biology, Clarkson University, Potsdam, NY, United States; ^5^Department of Nutritional Sciences, School of Public Health, University of Michigan, Ann Arbor, MI, United States; ^6^Division of Sleep Medicine, Department of Neurology, University of Michigan, Ann Arbor, MI, United States; ^7^Department of Environmental and Occupational Health, School of Public Health, Texas A&M University, College Station, TX, United States

**Keywords:** recommendations, physical health, mental health, older people, lower socioeconomic status

## Abstract

Low-income older adults are disproportionately impacted by the COVID-19 pandemic. In this perspective article, we review the context in which low-income older people experience the pandemic and the mental and physical health consequences they have faced to date. Then, we offer practical solutions to help improve low-income older adults' sleep, physical activity, nutrition, and stress that require no or low financial commitment. We argue that governments, communities, and organizations should make greater efforts to promote healthy living for low-income older adults in times of health emergencies to ensure their ability to be universally adopted, regardless of income and resources.

## Introduction

In many ways, the novel coronavirus disease 2019 (COVID-19) pandemic has raised awareness about the importance of public health and gerontology. It is now common knowledge that handwashing, mask wearing, and physical distancing are effective public health measures to help reduce the spread of infection. Lockdowns and visitor restrictions have also been implemented in an attempt to minimize hospitalizations and deaths from COVID-19, especially among older adults who have been the most at-risk (1). The adoption of these protective measures to mitigate the spread of COVID-19 have paradoxically resulted in unintended short- and long-term mental and physical health consequences for older adults (2–6). Thus, alongside efforts to prevent COVID-19 infection, disruptions in daily routines and lifestyle behaviors (e.g., physical activity, nutrition, sleep, social interactions) should not go overlooked. While there has been a surge in available resources and shifts in public health messaging to provide recommendations to older adults during the pandemic (7), specific recommendations are required for low-income older people (8, 9). Older adults with fewer financial resources and those who reside in more impoverished areas are often at a greater risk of COVID-19-related death (10, 11). Further, these older adults more frequently engage in unhealthy behaviors and have less access to healthful services, resources, and programs (12); which imposes barriers that limit their ability to self-manage their physical and mental health.

Herein, we present the results of our narrative literature review of the current knowledge on the mental and physical health of low-income older adults during the COVID-19 pandemic. For this perspective article, the literature review is not meant to be exhaustive, but aims to present an overview of recent literature about these key concepts (13). Through this narrative review, we contextualize the health consequences experienced by low-income older adults during the COVID-19 pandemic and offer practical recommendations to help them self-manage their physical and mental health. Using a socioecological perspective, we also provide public health recommendations beyond the individual level that could be implemented by governments, communities, and organizations for the well-being of low-income older adults.

## Context

In the United States, approximately 87% of adults ages 65 and older were retired in 2016 (14), relying on social security benefits, pensions, retirement savings accounts, savings, and supplemental security income as their main sources of income (14). Approximately 50 million social security beneficiaries are people 65 and older; social security contributes to 90% of the family income in 25% of households (14). In 2018, retired workers on social security received an annual income of $17,535 (15). Social security is not enough to cover out-of-pocket medical expenses (16), which may persuade patients to delay getting tested when they develop symptoms of COVID-19 or defer care and treatment in an attempt to avoid medical debt (17). If or by the time low-income older adults seek care, their condition may be worse with less chances of full recovery (18, 19). Uninsured or underinsured older Americans are at an increased risk of COVID-19 and its complications (20). Older African Americans are significantly more at risk of COVID-19 infections and mortality due to a variety of social determinants, including low income and lower rates of health insurance (21–23).

For older people who continue to work past retirement age, median earnings in 2018 were estimated at $35,036 (14). Job losses during the pandemic have been predominantly in occupations with the lowest weekly earnings, including in retail, leisure, and hospitality sectors, where older women, Black, Indigenous and people of color (BIPOC), and immigrant populations are highly employed (24, 25). According to the Congressional Research Service (14), between January and September 2020, 8% of older workers lost their jobs in the cleaning and maintenance sector, 10% in the food sector, and 28% in the personal care and service sector. BIPOC older adults working in sectors with high-contact, face-to-face interactions with the public may also choose to quit their jobs to reduce their risks of workplace exposure to COVID-19 (26).

The economic consequences of the pandemic have also contributed to greater rates of food insecurity among low-income households (27). In 2018, approximately 5.3 million older adults were food insecure (28); the number of food insecure older adults is expected to have significantly increased during the pandemic (28). Several reasons can explain this increase, including difficulty affording food (29), decline in food donations at food banks (30), trouble accessing food through a food pantry (29, 30), temporary closures of senior centers offering meals (30), challenges in getting food delivered by family or friends (29), less use of food delivery services or apps compared to other age groups due to related costs or access to technology (30), and fear of COVID exposure at the supermarket (30). Conversely, close to 20% of food-insecure adults are unable to buy 2 weeks of food at the same time to comply with the public health recommendations, thus increasing their risks of exposure to the virus (27).

## Mental Health Consequences

The impact on the mental health of low-income older adults has been widely reported. Adults with low socioeconomic position were found to be the most at risk of experiencing moderate to severe depressive symptoms during the pandemic (31). Another study revealed that those who perceive to be personally at risk of COVID-19, including low-income older adults, experience greater depression and anxiety (32). Low-income older adults who test positive for COVID-19 may suffer fear, stigma, and post-traumatic stress symptoms from their experiences (33–36). The general *marginalization* (37) of older people, requiring them to avoid intergenerational spaces (38), stay at home, self-isolate, and practice physical distancing from their families and friends, in addition to the digital divide (39), has contributed to further increasing the social isolation and loneliness of older people (37, 38, 40–45), which was deemed an epidemic prior to COVID-19 (46–48). In addition, low-income older adults may have less access to technology than other older adults, making it difficult for them to maintain their social connections (49). Marginalization of older people also encourages ageist speech, behaviors, and policies (50–52), resulting in negative consequences for the health of older people, including reinforcing depressive symptoms, loneliness, and premature death (51, 53). An increase in alcohol consumption has been noted during the pandemic (54); low-income older adults are at risk of turning to substances such as alcohol and drugs to cope with financial stress, loneliness and grief (41), among other reasons, which may increase their risks for suicide (55–57). Older adults with pre-existing mental health disorders may be more prone to relapse of substance misuse, social isolation, and suicidal behavior (58), especially due to limited access to mental healthcare services (58).

## Physical Health Consequences

The pandemic has also impacted the physical health of low-income older adults (59). Studies on COVID-19 around the world have reported a decrease in physical activity (4, 60–65) and an increase in sedentary behavior (i.e., sitting, reclining or lying down for long periods of time) (65–67) among older adults, which can significantly compromise an older person's cardiorespiratory fitness, muscle strength, and muscle mass (68, 69). Only 2 weeks of inactivity (e.g., 75% less steps in a day) can result in an 8% reduction in muscle strength (70); conversely, more than 2 weeks of rehabilitation would be needed for older people to regain their initial muscle function (70). Muscle deconditioning can accelerate the progression of sarcopenia (69), contributing to frailty, reduced mobility, and falls (4, 63, 71–73).

Other studies reported that a decrease in physical activity of just 1,500 steps per day can worsen blood glucose control (74–76), increase body inflammation (77) aggravate existing chronic diseases (e.g., diabetes) (74, 78, 79), and weaken the immune system (75), which may heighten an older person's risk to acute respiratory infections such as COVID-19 (75). Sedentary behavior is also associated with an increase in mental disorders such as depression and anxiety (80). Considering that low-income older adults are at greater risk for COVID-19, more prone to accelerated aging (81), chronic diseases, and disability (81, 82), and have reported poorer mental health during the pandemic (31) compared to the general older population, disrupting sedentary behavior and engaging in physical activity become especially important for this specific population (79, 83).

While malnutrition is generally considered to be an important issue for older adults (84, 85), it has continued to be a common occurrence during the pandemic (4). Older adults have experienced undernutrition, such as skipping meals due to food insecurity, as well as an overconsumption of unhealthy foods, such as sugar and saturated fats (4). Malnutrition can both increase the prevalence of chronic conditions (86) and complicate existing chronic diseases (87, 88). Poor nutrition can also impair the immune system and its defense against COVID-19 (89, 90). In fact, deficiencies in micronutrients such as vitamins A, C, D, zinc, and iron have been associated with adverse clinical outcomes related to COVID-19 (91). Nutrition, including adequate protein intake and vitamin D, is particularly important for low-income older adults during lockdowns and self-isolation to maintain muscle strength and balance (92) and prevent sarcopenia (93–95).

Low-income older adults are at an increased risk of sleep problems (96, 97). The pandemic situation has worsened older adults' sleep quality (42) and increased cases of insomnia (43). Older people with poor sleep quality also report greater levels of loneliness (42, 98), which have been linked to cardiovascular disease (99), dementia risk (100), poorer self-rated health (101), limited mobility (102), and premature death (47). General delays in seeking or obtaining medical care during the pandemic, including the cancellation of medical appointments for chronic disease care, may have also contributed to chronic disease complications and poor physical health of low-income older adults (43, 103, 104).

## Positive Consequences of the COVID-19 Pandemic for Low-Income Older Adults

While the negative consequences of the pandemic far outweigh the benefits, some positive repercussions can be noted, especially for low-income older adults. A study by Whitehead and Torossian described the joys experienced by low-income older adults during the pandemic, which included interactions with family/friends, digital communication, hobbies/entertainment, and pets (105). For low-income older adults living with a partner or confined with family members in multigenerational households, for example, the pandemic may have provided the opportunity to create stronger and more meaningful connections with one another (106, 107). Low-income older adults with access to technology may have gained more confidence using technology and online platforms that helped them maintain their social interactions and engage in hobbies, such as reading and listening to music (52).

It is also possible that the chronic stress experienced by low-income older adults throughout their lives may have helped them be more resilient during the pandemic, able to positively reframe the situation, and cope with these unusually stressful times (108–111). Some may have also turned to religion and spirituality as resources to manage their emotional and economic stress and find purpose and meaning during the pandemic (112).

## Individual-Level Recommendations for Healthy Living With No or Low Financial Commitment

In Table 1, we summarize practical recommendations for healthful lifestyles among low-income older adults during the COVID-19 pandemic. Many of these recommendations may be universal for older adults and individuals of all ages and may also apply outside of pandemic situations. However, they are particularly pertinent during the pandemic because they are feasible for individuals with limited resources. While these recommendations arose from the experience of the COVID-19 pandemic, they may apply more broadly during times when physical distancing is required or recommended for older adults, such as during annual flu seasons.

**Table 1:** Practical recommendations for low-income older adults.

**Topic**	**Strategies**
Sleep (113)	•Aim for 7–9 hours of sleep every night. •Establish a sleep schedule. It is important to maintain a consistent time for going to bed and waking up every day. This means avoiding napping in the late afternoon or evening before bedtime. Maintaining a sleep schedule will help the body establish a rhythm to help older adults fall asleep easier and remain asleep longer. •Establish a bedtime routine. Take time to decompress and relax before bed to increase your ability to fall asleep quickly. Reading a book or listening to music are great pre-bed routines. Avoid using technology (e.g., watching TV, using your smart phone or tablet) in bed to reduce over-stimulation. Additionally, avoid consuming large meals and/or caffeinated or alcoholic beverages in the hours before sleep as not to disrupt sleep quality and duration. •Create a conducive sleeping environment. Avoid unnecessary lighting and maintain a comfortable temperature that is neither too hot nor too cold. Minimize the exposure to sources of noise when possible. If sounds from traffic, housemates, or neighbors are unavoidable, consider earplugs or sources of white noise.
Physical activity (114–116)	•Break sedentary behavior every 20–30 minutes by walking or standing for 2–5 minutes. •Engage in 150–300 minutes a week of moderate-intensity physical activity. This can be done 10 minutes at a time, if needed, and can be as easy as taking a walk outside. Start slowly and build up your exercise time as you become more active. Also, consider stretching your muscles when they are warm. •Maintain your strength. Use your muscles as much as you can to avoid deconditioning that can increase your risk of falls. Strength training can be as simple as doing a few repetitions of bicep curls and overhead presses with soup cans or heavy water bottles. Moving is key so use the equipment that you have available at home. Avoid sitting down for long periods of time. •Practice keeping your balance. Balance and strength are important to prevent falls and fall-related injuries. You can train your balance by standing on one foot and then the other, or getting up from a chair without the support of your hands or arms. Go at your own pace and stay safe.
Nutrition (117)	•Stay hydrated. Make sure you drink plenty of water throughout the day. •Eat foods rich in nutrients. This includes foods like fruits and vegetables, whole grains, eggs, lean meats, fish, beans, and nuts. Maintain a high level of energy throughout the day by eating a few healthy snacks. Maintaining consistent eating times can be helpful for weight management. •Avoid foods filled with sugar, salt or saturated fat. Foods like chips, pastries, candy, ice cream, and soda contribute little nutritional value to your diet. •When possible, share meals with others. If you live with others, avoid dining alone. Invite others to eat with you or prepare a meal for the household so you can eat while enjoying each other's company. Avoid eating in front of a screen as much as possible so that you can take the time to enjoy the food you are eating.
Stress (118)	•Take care of yourself. Take some time during the day to take deep breaths. Slowly breathe in through the nose, focus on your breath as you let the air fill your belly, and then slowly exhale through your nose or mouth. Repeat a few times until you feel more calm or relaxed. •Make time to unwind at the end of every day. Engage in an activity that you enjoy such as reading a book, doing a puzzle, playing cards, calling your family or friends, or speaking with your neighbors while maintaining physical distancing. •Avoid consuming too much alcohol, tobacco, and other substances. These substances can aggravate symptoms of stress as well as increase the risk of developing substance use disorders.

## Recommendations for Governments, Communities, and Organizations

Governments, communities, and organizations can play important roles to improve healthy living among low-income older adults during the COVID-19 pandemic. Governments can implement policies that encourage healthy behaviors among low-income older adults (119). They can also prioritize and invest financial resources in populations (e.g., low-income older adults) or specific areas of deprivation (e.g., mental and physical health, internet access) (120). Established community networks, such as those from age-friendly communities (121, 122), can be leveraged to encourage organizations to work together across organizational and sector boundaries to meet the urgent needs of its low-income older adult populations. For example, the City of Lethbridge, Canada was recognized for this type of work where more than 50 organizations collaborated across sectors to fight food insecurity of low-income older adults during the pandemic (123). Government policies and investments can ultimately lead to greater funding for organizations so they can increase their availability, accessibility, and affordability of programs and services, including providing mental healthcare and well-being resources for low-income older adults (124).

## Discussion

In this perspective, we described the physical and mental health challenges associated with lifestyle disruptions caused by the COVID-19 pandemic. Individual-level recommendations for sleep, physical activity, nutrition, and stress provide a helpful framework for achieving healthy habits in spite of our changing society; however, meeting these guidelines may be especially challenging for older adults who are economically disadvantaged, those with limited family or social networks, and/or those experiencing physical, cognitive, or sensory impairments. These older adult subpopulations are not typically the focal group for health recommendations, despite being at higher risk for poorer health status based on financial barriers for healthy living. This may make meeting guidelines and adhering to recommendations impossible based on their available resources and economic position. Additional efforts and research are needed to refine and tailor guidelines for older adults in “pandemic living situations” to ensure their ability to be universally adopted, regardless of income and resources. As one example, while older adults may be eligible for and receive home-delivered meals, they may not have much control over the foods they receive from these programs. However, older adults may still be able to implement some of the healthy eating tips such as consistent meal times, eating with others, and not eating in front of a screen. As another example, given the need to remain physically distant from others to avoid the virus, the nation has turned to virtual and telephone solutions to engage older adults with community, social, and healthcare services. While these forms of “distanced connectivity” (2) may have value for older adults, the digital divide prevents many low-income older adults from accessing and benefiting from such services. Many lower-income and rural areas do not have high-speed broadband, and low-income older adults may not have access to computers, smartphones, or tablets regardless of internet access. As such, governments, communities, and organizations have important roles to support and promote healthy living among low-income older adults during the COVID-19 pandemic. As the world undergoes unprecedented changes, and disparities and inequities widen in terms of resource availability, it is increasingly critical to provide realistic health recommendations to low-income older adults to which they can reasonably adhere. As a society, we must implement system-level efforts to better support this population and complement their individual-level efforts for change.

## Data Availability Statement

The original contributions presented in the study are included in the article/supplementary material, further inquiries can be directed to the corresponding author/s.

## Author Contributions

All authors significantly contributed to the planning, drafting, and review of this manuscript.

## Conflict of Interest

The authors declare that the research was conducted in the absence of any commercial or financial relationships that could be construed as a potential conflict of interest.
